# Amelioration of Age-Related Multiple Neuronal Impairments and Inflammation in High-Fat Diet-Fed Rats: The Prospective Multitargets of Geraniol

**DOI:** 10.1155/2022/4812993

**Published:** 2022-10-18

**Authors:** Eman Fawzy El Azab, Shaymaa Abdulmalek

**Affiliations:** ^1^Clinical Laboratories Sciences Department, College of Applied Medical Sciences at Al-Qurayyat, Jouf University, Al-Qurayyat, 77454, Saudi Arabia; ^2^Biochemistry Department, Faculty of Science, Alexandria University, Alexandria 21511, Egypt

## Abstract

Neuroinflammation is documented to alter brain function as a consequence of metabolic changes linked with a high-fat diet (HFD). The primary target of this study is to see how geraniol is effective in manipulating age- and diet-associated multiple toxicity and neuroinflammation in HFD-fed rats. Sixty-four adult male Wistar rats were partitioned into two groups: Group 1 (untreated normal young and aged rats) and Group 2 (HFD-fed young and aged rats) that received HFD for 16 weeks before being orally treated with geraniol or chromax for eight weeks. The results revealed a dropping in proinflammatory cytokines (TNF-*α* and IL-6) and leptin while boosting adiponectin in geraniol-supplemented rats. The liver, kidney, and lipid profiles were improved in geraniol-HFD-treated groups. HFD-induced brain insulin resistance decreased insulin clearance and insulin-degrading enzyme (IDE) levels significantly after geraniol supplementation. Geraniol suppressed acetylcholinesterase (AChE) activity and alleviated oxidative stress by boosting neuronal reduced glutathione (GSH), catalase (CAT), glutathione-S-transferase (GST), and superoxide dismutase (SOD) activities. It lowered malondialdehyde concentration (TBARS), nitric oxide (NO), and xanthine oxidase (XO) and restored the structural damage to the brain tissue caused by HFD. Compared with model rats, geraniol boosted learning and memory function and ameliorated the inflammation status in the brain by lowering the protein levels of IL-1*β*, iNOS, NF-*κ*Bp65, and COX-2. In addition, the expression levels of inflammation-related genes (MCP-1, TNF-*α*, IL-6, IL-1*β*, and IDO-1) were lessened significantly. Remarkably, the supplementation of geraniol reversed the oxidative and inflammation changes associated with aging. It affected the redox status of young rats. In conclusion, our results exhibit the effectiveness of dietary geraniol supplementation in modifying age-related neuroinflammation and oxidative stress in rats and triggering off the use of geraniol as a noninvasive natural compound for controlling age- and diet-associated neuronal impairments and toxicity.

## 1. Introduction

Obesity is prevailing steadily worldwide, which negatively influences people's long-term health. It has been linked to insulin resistance (IR), hyperlipidemia, hypertension, type 2 diabetes (T2D), cardiovascular illness, and steatohepatitis [1]. Additionally, considerable evidence suggests that the most widespread disorders, such as metabolic pathologies related to obesity and neurodegenerative disorders, affect over 50% of the elderly population [2].

Inflammation is a stress response intended to aid adaptation and recovery from shocks [3]. An inflammatory state that lasts too long may impair cell processes, resulting in altered metabolism and pathological diseases [3]. Mitochondria, the primary generator of reactive oxygen species (ROS), produce critical components during inflammation and oxidation. Thus, mitochondrial dysfunction is linked to inflammation and other energy-related problems in which the body makes more ROS than it can protect with antioxidants, causing oxidative damage to cells [4].

Neuroinflammation in the brain is a significant risk factor for neurodegenerative illnesses characterized by learning and cognitive impairment due to disrupted neuronal connections and aberrant synaptic plasticity [5]. HFD triggers signaling pathways with negative consequences in multiple brain regions, including the hippocampus and brain cortex [6]. A relationship between human obesity and cognitive impairment has been documented [7]. Further, in the case of age-related cognitive decline, neuroinflammation and oxidative stress are known to be present in the hippocampus and may be linked to neurodegenerative impairments [8, 9]. Neuroinflammatory changes, including higher levels of cytokines in serum and CSF, grow with age, even in healthy brains [10]. These accumulating changes can be cytotoxic and disrupt essential neuronal functioning, leading to the emergence of neurodegenerative disorders [11]. Neuroinflammation has been observed in animal models even before amyloid accumulation [12].

The main origins of neuroinflammation may be correlated to the stimulus of a high-fat diet (HFD) and obesity on the inflammatory processes of neurons [13]. HFD may cause a systemic uptick in proinflammatory cytokines, such as tumor necrosis factor (TNF), interleukin 1 (IL-1), and interleukin 6 (IL-6), coupled with rising inflammatory signaling through the nuclear factor (NF-*κ*B) pathway, either peripherally or in the brain. These proinflammatory markers may be a direct source or a consequence of obesity, particularly IL-1*β*, IL-6, and TNF-*α*, which are associated with chronic and acute inflammation related to obesity [14]. These mediators' levels in the blood, along with CRP, are important damage biomarkers that link chronic metabolic illnesses like obesity and T2D to dementia or late-onset AD.

Dyslipidemia is another obesity-related condition that has been linked to an elevated risk of AD. High cholesterol consumption and hypercholesterolemia have been linked to neuroinflammation in animal models [15]. Furthermore, a lipid profile with high levels of low-density lipoprotein (LDL) cholesterol and low levels of high-density lipoprotein (HDL) cholesterol is linked to an increased risk of AD [16].

In the context of obesity, adipose tissue undergoes a functional switch, shifting the secretory profile of endocrine factors from homeostatic to proinflammatory, resulting in decreased metabolic flexibility [17] and increased levels of proinflammatory adipokines, resulting in a chronic low-grade inflammatory environment affecting the brain.

Inflammation and IR are two of the most frequent mechanisms underpinning the pathophysiology of obesity-related diseases. They are also present in several neuronal disorders [18]. The insulin-degrading enzyme (IDE, EC 3.4.24.56) is a zinc metallopeptidase that degrades a variety of physiological peptides, including insulin and amyloid-peptide (A). It is also known as insulin or insulin protease. The proteolytic action of IDE on insulin and its dysregulation have been commonly scrutinized in T2D [19]. However, given its degradative capacity for amyloidogenic peptides, such as A*β*, and its aggregation suppression activity over amyloidogenic proteins, it was investigated as a significant regulator of diseases like Alzheimer's and Parkinson's disease [20]. The brain has been thought to be an insulin-responsive organ, and IDE has been highlighted as an important function in the breakdown and clearance of insulin. Moreover, higher expression of IDE in cultured cells has been reported to speed up insulin breakdown. However, injecting cells with IDE-specific antibodies delay insulin breakdown [21].

Herbal medicines have proven efficient pharmacological therapy as potential alternative therapeutic agents against neurodegeneration due to their neuroprotective benefits and lack of adverse effects compared to new synthetic medications [22]. Geraniol (E-3,7-dimethyl-2,6-octadien-1-ol) is an acyclic monoterpene found in different plant species. It is a vital component of the essential oils of cardamom, ginger, orange, lime, lemon, nutmeg, lavender, and rose and the principal component of orange flower and palmarosa oils [23]. According to a previous study, geraniol has considerable pharmacological activity, including antioxidant, antimutagenic, and anti-inflammatory characteristics. Furthermore, it inhibits tumor growth in murine leukemia, hepatoma, and melanoma cells [24]. Valdes et al. [25] proved that geraniol suppresses glucose absorption by repressing the *α*-glucosidase enzyme and sodium-glucose cotransporter (SGLT-1).

Therefore, the present study is aimed at highlighting how naturally occurring geraniol may affect age- and diet-related cognitive decline, neurotoxicity, and IR through the molecular pathways and genes' expression associated with neuroinflammation prompted by HFD in young and aged male Wistar rats, along with evaluating geraniol's potential to repair the metabolic and physiological imbalances caused by HFD and its effect on learning and memory functions, to support our rationale for using natural resources to protect age- and diet-related complications in the brain.

## 2. Material and Methods

### 2.1. Ethics Statement

All experimental procedures were permitted by the Ethical Committee of the Faculty of Science, Alexandria University, Egypt (AU 04210403302), and were conducted in keeping with the regulations provided by Alexandria University's Animal Protocol Guidelines. The discomfort and suffering experienced by the animals were kept to a minimum.

### 2.2. Animals

In the present study, 64 male Wistar rats were used. The Animal House, Institute of Graduate Studies and Research, Alexandria University, Egypt, provided 32 Wistar young rats (4 months) and 32 Wistar old rats (20 months). The rats were hosted in transparent solid-bottom, polycarbonate cages with stainless-steel wire lids (4 rats/cage). They were retained in a maintenance facility in the Institute of Graduate Studies and Research in a room with a 12 h light/12 h dark cycle, a controlled temperature of 22 ± 2.0°C, and controlled humidity of 45%-46% with water-free access and stable normal pellet diet.

### 2.3. Experimental Design

**Figure 1:**
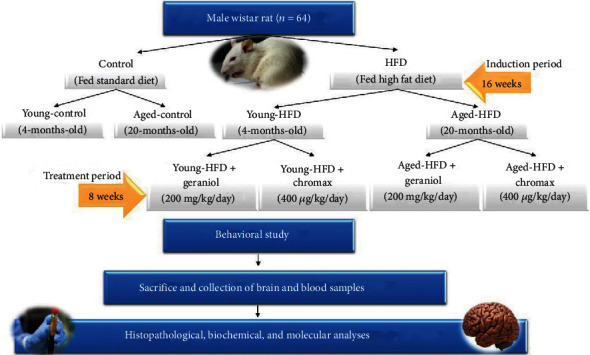
Group classification and study design.

The main target of this research is to examine how geraniol may affect age- and diet-related rise in neuroinflammation, as well as other related complications. Since obesogenic diets with high-calorie content remain a key factor in obesity induction, we started the study using young rats (4 months) and aged rats (20 months) and fed them HFD for 16 weeks. Following acclimatization, young and aged rats were classified into two major groups as follows: Group 1 (normal young and aged rats) served as untreated normal control, receiving a normal pellet diet (NPD) including (26% protein, 3% fat, 54% carbohydrate, and 17% vitamins and minerals percentage per 100 gm), and Group 2 (HFD-fed young and aged rats) received HFD (including protein 25%, fat 58%, and carbohydrate 17%, as a percentage of total kcal) for 16 weeks and then orally treated with geraniol (Sigma-Aldrich, St. Louis, MO, USA) with a dose of 200 mg/kg body weight (BW) or chromax, as a standard antiobesity drug with a dose of 400 *μ*g/kg BW mixed in normal saline once per day for eight weeks with free access to water (Figure 1). The dose of the geraniol was chosen based on the earlier literature [26]. Geraniol was mixed in normal saline and vigorously vortexed each time before administration to rats through the oral gavage. Food and water consumption, along with the performance of rats, was monitored daily. The body weight of rats was assessed weekly from the beginning of the experimental period. After the treatment period, overnight fasting animals were weighed and anesthetized, blood was collected using a syringe puncture from the abdominal aorta, and serum was isolated. The brain tissues were quickly removed, washed with ice-cold 0.9% NaCl, frozen in liquid nitrogen, and kept at -80°C for further analyses. The remaining part of the brain was kept in formalin (10%) for histopathological study.

### 2.4. Biochemical Parameters

Serum levels of liver parameters, such as aspartate aminotransferase (AST) and alanine aminotransferase (ALT), alkaline phosphatase (ALP), albumin (ALB), total protein, and total bilirubin; kidney biomarkers; creatinine and urea levels and lipid profile; and low-density lipoprotein (LDL), high-density lipoprotein (HDL), total cholesterol (TC), and triacylglycerol (TG) were assessed using standard colorimetric kits (Spectrum Diagnostics, Egypt) as stated by the manufacturer's guidelines. In addition, serum insulin concentrations were assessed using a rat insulin ELISA kit (MyBioSource, MBS724709). Insulin sensitivity was evaluated using the homeostatic model assessment (HOMA-IR) index, which was calculated as plasma glucose (mg/dl) × fasting plasma insulin (IU mg/l during fasting state divided by 405) [27].

### 2.5. *In Vivo* Insulin Clearance

For intraperitoneal insulin tolerance tests, rats fasted for 3 h. Then, they received a 1 U/kg insulin intraperitoneally (i.p.). Insulin levels were measured before (0 min) and 5, 15, 30, and 60 min after insulin administration, and the area under the curve was calculated [28].

### 2.6. ELISA Assessment of Pplasma C-Peptide and Brain IDE Levels

The levels of plasma C-peptide level (MyBioSource, MBS704133) and brain insulin-degrading enzyme (IDE) level (MyBioSource, MBS2516115) were measured using a rat-specific ELISA kit, following the manufacturer's protocol.

### 2.7. Morris Water Maze Test

To examine spatial memory, the Morris water maze (MWM) test was used [29]. Water was filled to a depth of 25 cm in a black circular pool (180 cm in diameter and 60 cm in height) at 23 ± 1°C. Four quadrants were created in the pool: northeast (NE), northwest (NW), southeast (SE), and southwest (SW). Each animal was gently taken out of the water, placed in a heated holding cage, and returned to its home cage after each training and testing. In the northern quadrant's center, a black escape platform was submerged 1 cm below the water level. The animals were placed in water at one of four randomly chosen sites, and the time between entering the water and escaping onto the platform (escape latency) was recorded. For four days, animals were taught with two blocks of four 60 s trials at around the same time daily. The animals spent 30 s on the platform between each trial and were given a 5 min break between the two blocks. If one did not succeed in locating the platform within 60 s, the researchers gently returned it to the platform and allowed it to stay there for 30 s. The escape latency, length calculated for the swim path (distance traveled), and time spent within the target quadrant were recorded. Each rat completed a single 60 s probe trial and a visible platform trial on the fifth day. For the probe trial (MWM day 8), the testing environment was an exact match for the hidden platform task, but no platform was placed in the pool. During this one-time trial, every animal in the group was to swim in free manners for 60 s. Spatial memory was analyzed by measuring the time spent by the animals in the target quadrant.

### 2.8. Brain ELISA Immunoassay for the Quantification of Inflammatory Parameters

In preparation for assays, frozen brain tissue was homogenized in lysis buffer (150 mM NaCl, 10 mM Tris solution, 1% Triton X-100, pH 7.4), including protease inhibitor (Sigma-Aldrich, St. Louis, MO, USA), subsequently centrifuged at 13,000 rpm at 4°C for ten min, and the supernatant was collected. Then, the total protein content of the brain homogenate was analyzed by a previously reported protein assay [30]. ELISA levels of IL-6, inducible nitric oxide synthase (iNOS), and tumor necrosis factor-alpha (TNF-*α*) (MyBioSource; MBS355410, MBS263618, and MBS2507393, respectively) were assessed using commercial rat-specific ELISA kits following the manufacturer's guidelines.

### 2.9. Assessment of Obesogenic Diet-Related Hormones and Biomarkers in the Serum of Rats

The levels of serum leptin (MyBioSource, MBS012834) and adiponectin (MyBioSource, MBS068220) were measured using a rat-specific ELISA kit, following the manufacturer's protocol.

### 2.10. Preparation of Brain Samples and Investigation of Oxidative-Related Biomarkers

Frozen brain tissue was homogenized in lysis buffer (150 mM NaCl, 10 mM Tris solution, 1% Triton X-100, pH 7.4), including protease inhibitor (Sigma-Aldrich, St. Louis, MO, USA), thereafter centrifuged at 13,000 rpm for 10 min at 4°C. The supernatant was collected. The total protein content of the brain homogenate was determined using the previously mentioned standard procedure [30]. The thiobarbituric acid reaction (TBARS) method was applied to calculate lipid peroxidation levels and malondialdehyde (MDA) in brain homogenate. MDA values were stated as nmol/mg protein, and the pink chromogen was measured at 532 nm [31]. The brain nitric oxide (NO) level was valued using the Griess reagent method, in which the developed pink color was determined at 520 nm, and brain NO was stated as *μ*M/mg protein [32]. The activity of xanthine oxidase (XO) in the brain was estimated as designated before and expressed as *μ*mol/h/mg protein [33]. As explained previously, reduced glutathione (GSH) levels in the brain homogenate were calculated. After the formation of the yellow color, the absorbance was immediately measured at 412 nm, and the GSH levels were stated as mmol/mg protein [34]. Catalase (CAT) activities were assessed using a previous method in which the changes in absorbance were determined after 1 min at 240 nm, and CAT activity was expressed as U/min [35]. The activity of glutathione-S-transferase (GST) was assessed in the brain at 310 nm, and the specific activity of GST was expressed as *μ*mol/min/mg protein [36]. A previous method was utilized to evaluate superoxide dismutase (SOD) activity in brain homogenates; the absorbance was estimated at 420 nm, and the sample enzyme activity was stated in U/mg protein [37].

### 2.11. Quantification of Acetylcholine Esterase (AChE) Activity

The activity of AChE was quantified by Ellman's reagent colorimetric assay [38], as previously mentioned. It was assessed using 0.75 mM acetylthiocholine and 0.5 mM 5,5-dithiobis(2-nitrobenzoic acid) (DTNB) in 5 mM HEPES buffer (pH 7.5) (Sigma-Aldrich, St. Louis, MO, USA) at 412 nm.

### 2.12. RNA Isolation from the Brain, cDNA Formation, and Quantitative RT-PCR

RNA was extracted from brain tissue, using a standard method of the QIAzol Lysis Reagent (Qiagen, 79306). The SensiFAST cDNA Synthesis kit (BIO-65054) for RT-PCR was then used to synthesize cDNA from total RNA using the reverse transcription process. Finally, RT-PCR amplification of cDNA was carried out using the SensiFAST SYBR Green No-ROX Kit (BIO-98005). PCR cycling conditions were initiated by the activation of Taq DNA polymerase at 94°C, 15 min, followed by 40 cycles of denaturation at 95°C, 15 s; annealing at 60°C, 30 s; and final extension at 72°C, 30 s. Relative gene expression was measured in triplicate [39]: GAPDH (NM_017008.4) sense: 5′-TGCACCACCAACTGCTTAG-3′, antisense: 5′-GGATGCAGGGATGATGTTC-3′; MCP-1 (monocyte chemoattractant protein-1) (M57441.1) sense: 5′-CATGCTTCTGGGCCTGCTGTTC-3′, antisense: 5′-CCT GCT GCT GGT GAT CCT CTT GTA G-3′; TNF-*α* (XM_039105873.1) sense: 5′-GCC AGC CTC CGA AGC CAG C-3′, antisense: 5′-GGG CGG TAG CGT CCT TGG G-3′; IL-1*β* (NM_212844.2) (forward: 5′-TGG ACT TCG CAG CAC AAA ATG-3′; reverse: 5′-GTT CAC TTC ACG CTC TTG GAT-3′); IL-6 (NM_031168.2) (forward: 5′-CCA GAA ACC GCT ATG AAG TTC CT-3′; reverse: 5′-CAC CAG CAT CAG TCC CAA GA-3′); and IDO (indoleamine 2,3-dioxygenase) (XM_024929988.2) (forward: 5′-AGA AGT GGG CTT TGC TCT GC-3′; reverse: 5′-TGG CAA GAC CTT ACG GAC ATC TC-3′).

### 2.13. Western Blot Analysis

The frozen brain was homogenized using specific lysis buffer (100 mM EDTA, 100 mM NaCl, 0.5% Na-deoxycholate, Nonidet p-40, 10 mM Tris, and pH 7.5 with protease inhibitors). Samples were centrifuged at 12,000 rpm for 20 min at 4°C, supernatants were collected, and protein concentrations were measured using the Bradford assay. The brain samples (40 *μ*g) were boiled in SDS-PAGE sample buffer, separated on SDS-PAGE gels (10%), and then transferred onto a nitrocellulose membrane [40]. Blots were incubated in blocking buffer for 2 h at room temperature, washed with TBST buffer, and incubated overnight at 4°C with primary antibodies (Cell Signaling Technology, Beverly, MA, USA); COX-2 (12282), iNOS (13120), IL-1*β* (31202), NF-*κ*Bp65 (8242), and *β*-actin (4967). Then, the blots were incubated with a secondary antibody after washing for 2 h at room temperature. The bands were visualized with an alkaline phosphatase-specific kit (ab83369), and the bands were quantified using Image Lab 6.1 software (Bio-Rad).

### 2.14. Histopathology Study

Brain tissues from all groups were fixed in 10% formalin, then dehydrated using alcohol, and fixed in paraffin. Using a rotatory microtome, sections were cut (3 *μ*m thick) and mounted on clean glass slides, then stained using hematoxylin and eosin (H&E), and investigated under a light microscope [41].

### 2.15. The Semiquantitative Grading System for Brain Histopathological Alterations

Concisely, five random fields from each animal brain histopathological section were studied (×100). The grade of the identified lesion severity was evaluated depending on the percentage of the affected area/entire section and recorded as follows: (-): absence of lesion, (+): for a mild degree of lesions (5–25%), (++): for moderate lesion degree (26–50%), and (+++): for a severe degree of lesions (≥50%).

### 2.16. Statistical Analysis

ANOVA was used to explore group comparisons, followed by Scheffe post hoc studies. Statistical studies were conducted using IBM SPSS statistics 25 software (International Business Machines Corporation, IBM, USA). Statistical significance was set at *p* < 0.01, and all results were expressed as the mean ± SEM.

## 3. Results

### 3.1. Effect of HFD on Weight in Young and Aged Rats

**Figure 2:**
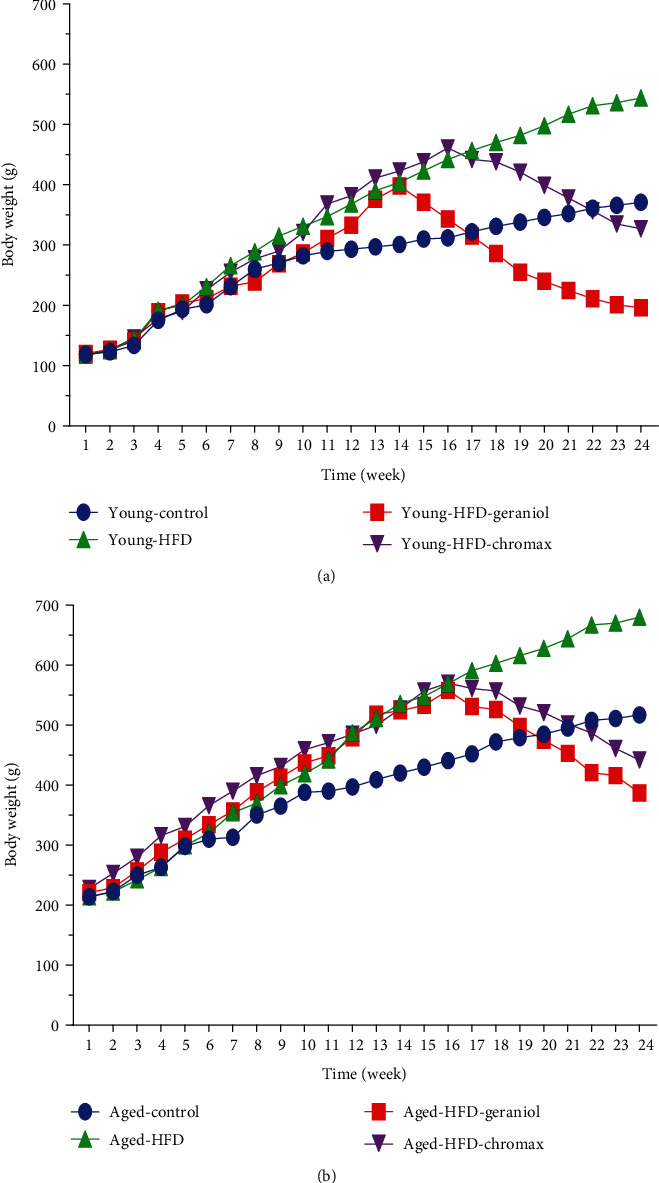
Body weight alterations in all studied groups: (a) body weights of young rats expressed as grams and (b) body weights of aged rats expressed as grams. Values are expressed as the mean ± SEM (*n* = 8).

An obese rat model was successfully developed after 16 weeks of HFD feeding, as evidenced by the obvious increase in body weight in the young and old HFD-fed groups compared to the control NPD-fed group. The weekly changes in body weight levels in the control and experimental groups are shown in Figure 2. Figures 2(a) and 2(b) show an overall effect of age, with older HFD-fed rats gaining weight quicker than younger HFD-fed rats. During the treatment period, geraniol or chromax administration resulted in a decrease in body weight as a function of age. The additional geraniol or chromax had various effects on the different groups. Body weight fell in young HFD-fed rats treated with geraniol more than in older HFD-fed rats, as well as chromax-treated rats at the end of the treatment period, which was comparable to the HFD-fed group.

### 3.2. Effect of Geraniol on Serum Liver, Kidney, and Lipid Profiles along with Insulin and Insulin Sensitivity Levels

As presented in Tables 1(a) and 1(b), young and aged rats fed HFD showed significantly higher serum levels of lipid profile, LDL, cholesterol, and TG along with low HDL levels, with increased liver functions, ALT, and AST and increased kidney functions, urea, and creatinine than control NPD-fed rats. In contrast, the daily administration of geraniol or chromax caused normalization of serum biomarker levels in both young and aged HFD-fed rats compared to control NPD-fed rats (*p* < 0.01, Tables 1(a) and 1(b)).

The results of fasting glucose levels revealed a significant effect on diet and age. In the control NPD-fed rats, glucose levels did not vary with age. Compared to the values of young HFD-fed rats, glucose levels in the aged HFD-fed group were significantly increased and remarkably increased from the same age in the control NPD-fed group (Table 2). The administration of geraniol exhibited a hypoglycemic effect displayed by a noteworthy decrease in blood glucose levels in both young and aged HFD-fed rats compared to HFD-fed rats.

The results of the analysis of insulin levels exhibited a significant effect on diet and age. They showed that the insulin levels did not alter with age in the control NPD-fed rats, but they augmented significantly with age in the HFD-fed rats compared to young HFD-fed rats. They were also markedly different from the corresponding age in the NPD-fed rats. We noticed that the daily administration of geraniol or chromax caused the normalization of serum insulin levels in both young and aged HFD-fed rats (*p* < 0.01, Table 2). Also, the obtained results from the homeostatic model assessment (HOMA) index exhibited a significant effect on diet and age. In the HFD-fed rats, the HOMA index elevated progressively with age compared to young HFD-fed rats and was markedly different from the corresponding age in the control NPD-fed rats. At the end of 16 weeks, young and aged HFD-fed rats showed a significantly higher HOMA-IR than the control NPD-fed rats. The daily administration of geraniol or chromax significantly ameliorated these changes in aged HFD-fed rats. Moreover, the normalization of HOMA-IR was only seen in young HFD-fed rats compared to control NPD-fed rats (*p* < 0.01, Table 2).

### 3.3. Effects of Geraniol on Plasma C-Peptide and Brain IDE Levels of HFD-Fed Rats

As presented in Table 2, we found that the C-peptide levels were affected by age and diet in rats; feeding rats with HFD for a long time significantly increased C-peptide levels in young and aged HFD-fed rats compared to the control NPD-fed rats. This upsurge in C-peptide was associated with the elevation in insulin levels as they were cosecreted in plasma. In contrast, C-peptide levels were ameliorated significantly following the treatment with geraniol or chromax for eight weeks in both young and aged HFD-fed rats concerning the control NPD-fed rats. The IDE level in the brain was studied to explain the impaired insulin clearance in HFD-fed rats. Compared to control NPD-fed rats, aged HFD-fed rats had a lower neural IDE level than young HFD-fed rats (Table 2). In comparison to untreated rats, geraniol treatment boosted neural IDE levels in HFD-fed rats.

### 3.4. Geraniol Normalized Insulin Clearance in HFD-Fed Rats

**Figure 3:**
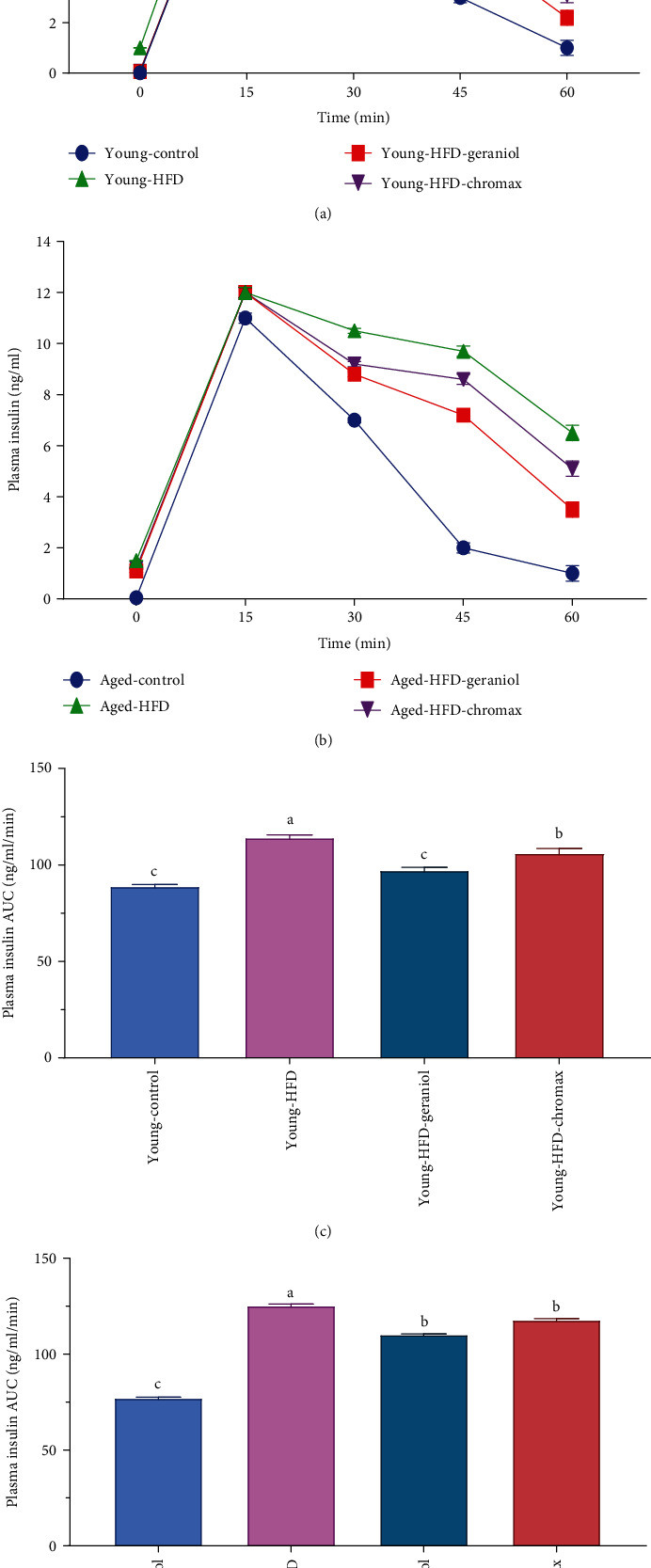
HFD effects on insulin clearance in rats: (a) plasma insulin at 0, 5, 15, 30, and 60 min of young rats; (b) plasma insulin at 0, 5, 15, 30, and 60 min of aged rats; (c) plasma insulin AUC of young rats; (d) plasma insulin AUC of aged rats post i.p. injection of insulin (1 U/kg). Values are expressed as the mean ± SEM (*n* = 8); means with different letters in each bar (A–D) are significantly different (*p* < 0.01), where the largest data value takes the letter (A) and the smallest data value takes the letter (D).

In our experiment, we measured insulin clearance in all groups of rats since insulin removal is an important component in regulating plasma insulin levels. Insulin clearance declined in aged HFD and young-HFD-fed rats compared to control NPD-fed rats, as illustrated in Figures 3(a) and 3(b). The area under the curve (AUC) showed that geraniol administration corrected insulinemia in HFD-fed rats, demonstrating enhanced insulin clearance in these animals. The young HFD-fed rats, in particular, had a more efficient rise in insulin clearance than the aged HFD-fed rats treated with geraniol or chromax, with the geraniol-treated rats having a more significant effect, as shown in Figures 3(c) and 3(d).

### 3.5. Effects of Geraniol on the Performance of Morris Water Maze (MWM) in HFD-Fed Rats

**Figure 4:**
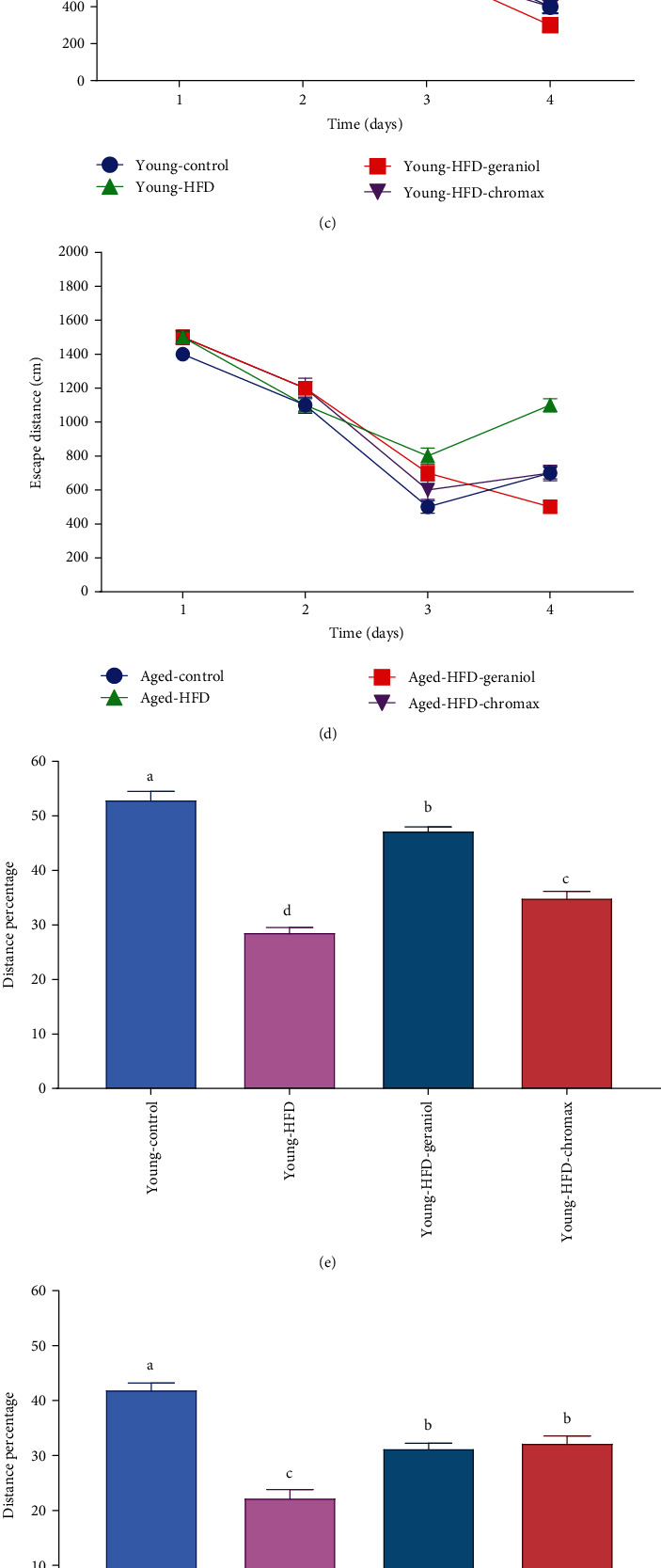
Effects of geraniol on memory functions in the brain of studied groups: the Morris water maze (MWM) was used to evaluate (a) escape latency for young rats, (b) escape latency for aged rats, (c) escape distances for young rats, (d) escape distances for aged rats, (e) the percentage of time spent for young rats, (f) the percentage of time spent for aged rats, (g) distance swum in the target quadrant for young rats, and (h) distance swum in the target quadrant for aged rats. Three independent experiments were carried out. Values are expressed as the mean ± SEM (*n* = 8). Means with different letters in each bar (A–D) are significantly different (*p* < 0.01), where the largest data value takes the letter (A) and the smallest data value takes the letter (D).

MWM assessed learning and memory functions with the hidden platform task. The MWM test was used to measure how long it took rats to recognize a water maze platform (escape latency) and the total swimming distance (escape distance). For the first three days, there was no significant difference in escape latency (Figures 4(a) and 4(b), *p* < 0.01) or distance (Figures 4(c) and 4(d), *p* < 0.01) between groups. The aged HFD group, in contrast, had considerably greater escape latency and distance than the young HFD- and NPD-fed rats on the fourth day (*p* < 0.01). Furthermore, HFD-fed rats receiving geraniol had considerably depressed the escape latency and distance (*p* < 0.01) compared to untreated rats. However, young HFD rats given geraniol had significantly longer escape latency and distance than aged HFD rats given geraniol (*p* < 0.01). Probe testing revealed that the aged HFD-fed group had considerably shorter swim time (Figures 4(e) and 4(f)) and distance swum (Figures 4(g) and 4(h)) in the target quadrant (represented as a % of distance swum and total time) than the control NPD-fed and young HFD-fed rats (*p* < 0.01). Treatment groups had considerably higher percentages of time and distance swum than untreated rats (*p* < 0.01). However, young HFD-fed rats given geraniol for eight weeks spent significantly more time and swam significantly more distance than aged HFD-geraniol-treated rats (*p* < 0.01).

### 3.6. Geraniol Alleviates the Modifications in Brain Inflammatory Markers in Aged and Young Rats Fed with HFD

**Figure 5:**
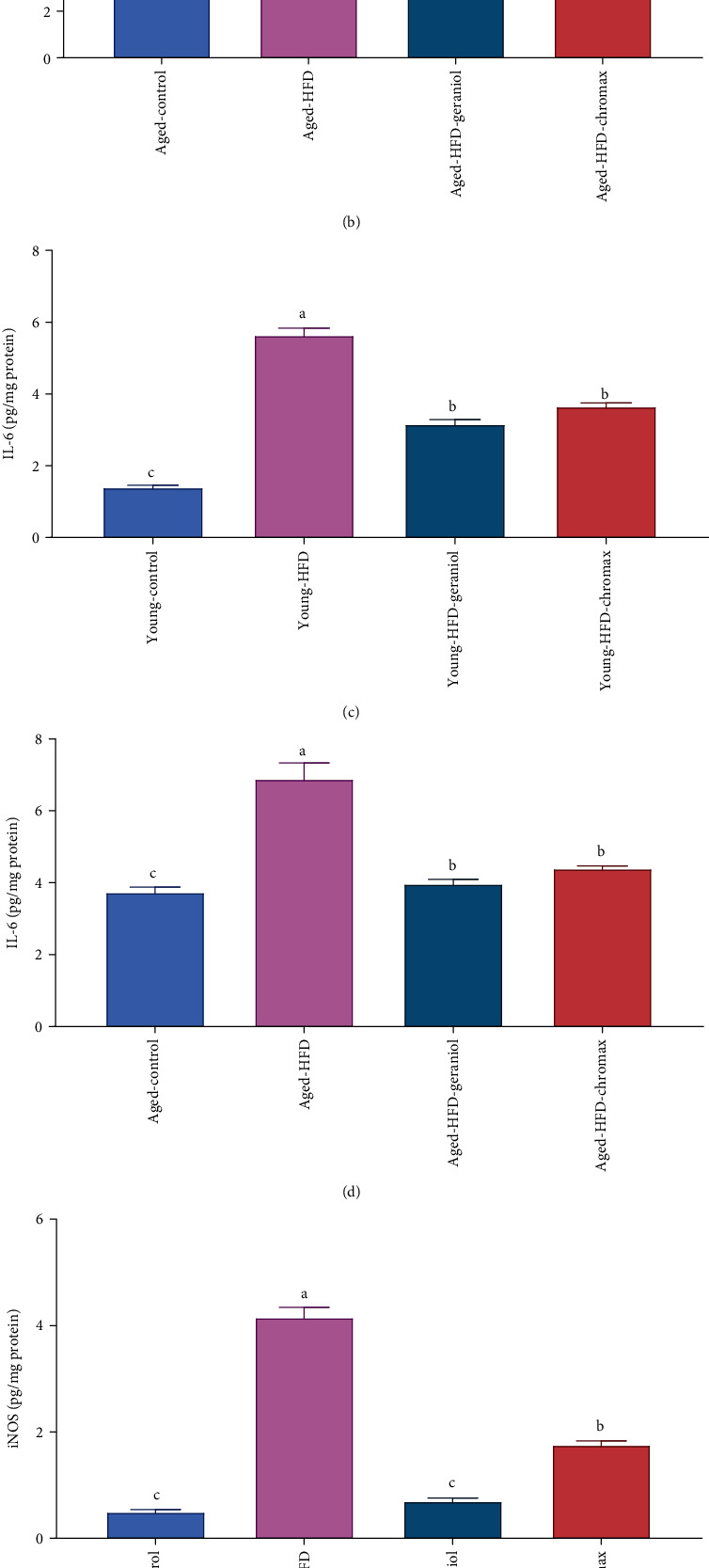
Effect of geraniol on neuroinflammatory response in studied groups: (a) TNF-*α* levels of young rats, (b) TNF-*α* levels of aged rats, (c) IL-6 levels of young rats, (d) IL-6 levels of aged rats, (e) iNOS levels of young rats, and (f) iNOS levels of aged rats. Values are expressed as the mean ± SEM (*n* = 8). The means of the same measured parameter with different letters in each bar (A–D) are significantly different (*p* < 0.01), where the largest data value takes the letter (A) and the smallest data value takes the letter (D).

As displayed in Figure 5, the levels of neuroinflammatory markers, including TNF-*α* (Figures 5(a) and 5(b)), IL-6 (Figures 5(c) and 5(d)), and iNOS (Figures 5(e) and 5(f)) were expressively affected by diet and age. The existing findings exhibited that the neural inflammatory cytokine levels elevated significantly with age in aged control NPD-fed rats than in young control NPD-fed rats. Also, the levels of the inflammatory parameters augmented significantly in aged HFD-fed rats concerning those in young HFD-fed rats. They were markedly different from the corresponding age in the control NPD-fed rats. Conversely, the administration of geraniol for eight weeks displayed a significant alleviation in inflammatory marker concentrations in all treated groups, especially in young HFD-fed rats than aged HFD-fed rats compared to control NPD-fed rats. The results also revealed that geraniol supplementation had a more potent effect than chromax on young and aged HFD-fed rats.

### 3.7. Geraniol Modulates Hormone Levels in HFD-Fed Rats

**Figure 6:**
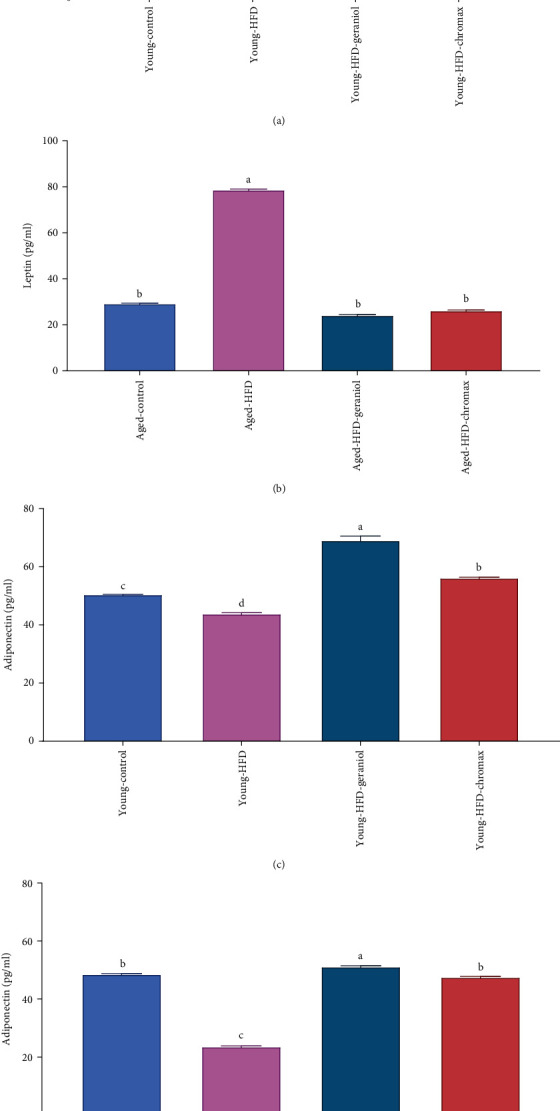
Effect of geraniol administration on levels of serum leptin and serum adiponectin in studied groups: (a) leptin levels of young rats, (b) leptin levels of aged rats, (c) adiponectin levels of young rats, and (d) adiponectin levels of aged rats. Values are expressed as the mean ± SEM (*n* = 8). Means with different letters in each bar (A–C) are significantly different (*p* < 0.01), where the largest data value takes the letter (A) and the smallest data value takes the letter (C).

The results of leptin and adiponectin concentration in the serum displayed a marked effect on diet and age. Adiponectin and leptin did not noticeably alter with age in the control NPD-fed rats. In contrast, HFD-fed rats demonstrated a noticeable elevation in leptin (Figures 6(a) and 6(b)) along with a marked reduction in adiponectin levels (Figures 6(c) and 6(d)) when compared to the values taken of young HFD-fed rats and also from the values of corresponding age in the control NPD-fed rats. The administration of geraniol for eight weeks ensued in strong depletion of leptin concentration with a marked increase in adiponectin levels in young HFD-fed rats than in aged HFD-fed rats. Nevertheless, a marked amelioration was observed in leptin and adiponectin concentrations in young HFD-treated rats with geraniol than the HFD-chromax-treated group for both young and aged rats.

### 3.8. Geraniol Alleviates Oxidative Damage in the Brain of HFD-Fed Rats

The effects of a 16-week HFD diet and an 8-week geraniol supplementation on oxidative damage mediators in the brains of young and old rats were investigated. TBARS, XO, and NO concentrations were assessed to observe the power of geraniol supplementation *in vivo* (Table 3). The MDA, NO, and XO concentrations in the brain tissue displayed significant effects on diet and age. MDA, NO, and XO concentrations increased but did not significantly change with age in the control NPD-fed groups. Otherwise, in the aged HFD-fed group, MDA, NO, and XO levels were substantially improved when compared with the values of young HFD-fed rats and markedly increased from the corresponding age in the NPD-fed group. In contrast, the magnitude of the tested drug effects differed significantly. The administration of geraniol or chromax to HFD-fed rats for eight weeks noticeably dropped in the oxidative stress in the brain of young-HFD fed rats more than aged HFD-fed rats compared to untreated rats, with a strong effect of geraniol than chromax.

### 3.9. Geraniol Alleviates Neural AChE Activity in HFD-Fed Rats

AChE activity in the brain tissue displayed a significant effect on diet and age. By comparing the aged and young rats, we observed that key AChE activity increased significantly in the aged HFD-fed rats than in the young HFD-fed group. This elevation was markedly different from the corresponding value in the control NPD-fed and young HFD-fed rats (*p* < 0.01). Compared with HFD-fed untreated rats, simultaneous treatment with geraniol significantly decreased AChE activity in the brain of young and aged HFD-fed rats, with more effective results in young HFD-fed rats treated with geraniol than in the chromax aged HFD-fed group (Table 3).

### 3.10. Geraniol Enhances Antioxidant Capacity-Related Biomarkers in the Brain of Rats

The upshot of age and diet on enzymatic, GST, CAT, and SOD along with nonenzymatic, GSH antioxidants in aged and young rats was estimated (Table 4). Antioxidant capacity in the brain of normal rats did not change significantly with age. While GST, CAT, and SOD activities besides GSH level in the HFD-fed group were substantially depressed in aged HFD-fed rats correlated to the young-HFD fed rats and decreased significantly compared to corresponding age in the control NPD-fed group. The eight-week treatment with geraniol instigated a noteworthy increase in SOD, CAT, and GST activities besides GSH levels in both young and aged HFD-fed rats compared with control NPD-fed groups. Statistically, the young HFD-fed rats exhibited a more effective amelioration of antioxidant capacity in the brain than in the aged HFD-fed rats treated with geraniol. Interestingly, geraniol treatment demonstrated a more significant increase in antioxidant levels than chromax in both aged and young rats (Table 4).

### 3.11. Effect of Geraniol on the Expression of Genes in Brain Tissue Involved in Inflammation

**Figure 7:**
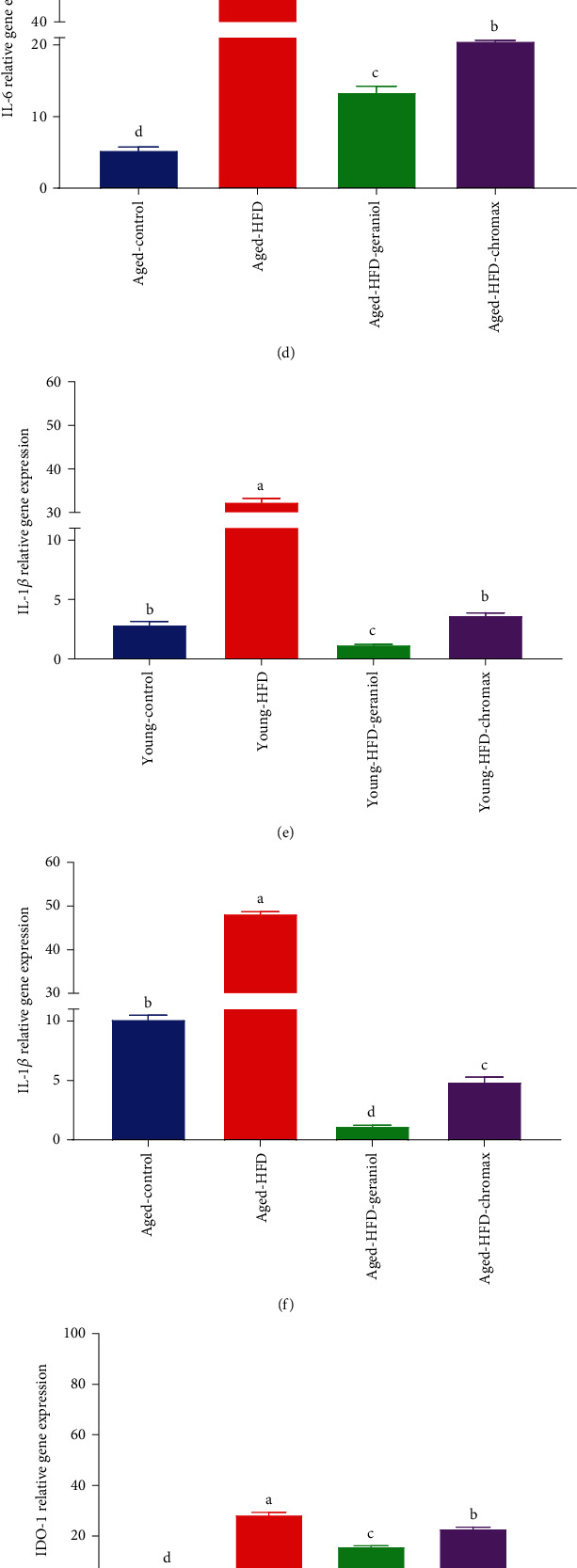
Gene expression profile of neuroinflammatory markers in all studied groups: (a) TNF-*α* expression in the brain of young rats, (b) TNF-*α* expression in the brain of aged rats, (c) IL-6 expression in the brain of young rats, (d) IL-6 expression in the brain of aged rats, (e) IL-1*β* expression in the brain of young rats, (f) IL-1*β* expression in the brain of aged rats, (g) IDO-1 expression in the brain of young rats, (h) IDO-1 expression in the brain of aged rats, (i) MCP-1 expression in the brain of young rats, and (j) MCP-1 expression in the brain of aged rats. Values are expressed as the mean ± SEM (*n* = 3). Means of the same measured parameter with different letters in each bar (A–D) are significantly different (*p* < 0.01); the largest data value takes the letter (A), and the smallest data value takes the letter (D).

Brain samples from each group of animals were subjected to mRNA expression analysis. Considerable changes induced by the HFD were observed in the neuroinflammation-related biomarkers. The results of TNF-*α* (Figures 7(a) and 7(b)), IL-6 (Figures 7(c) and 7(d)), IL-1*β* (Figures 7(e) and 7(f)), IDO (Figures 7(g) and 7(h)), and MCP-1 (Figures 7(i) and 7(j)) expressions in the brain displayed significant effects on diet and age. TNF-*α*, IL-6, IL-1*β*, IDO, and MCP-1 expression levels did not significantly change with age in the aged and young control groups. In contrast, in the HFD-fed group, they upregulated significantly with age. Comparing the aged and young rats, we found key genes of neuroinflammation TNF-*α*, IL-6, IL-1*β*, IDO, and MCP-1 expression levels to be upregulated in aged HFD-fed rats more than young HFD-fed group. This increase was significantly different from the corresponding value in the control NPD-fed group. In comparison, the administration of geraniol for eight weeks resulted in strong downregulation of gene expression of inflammatory markers with marked depletion in young HFD-fed rats than aged HFD-fed rats with a more potent effect of geraniol-treated rats than chromax-treated rats for both young and aged rats.

### 3.12. Geraniol Ameliorates Neuroinflammatory Protein Expression in the Studied Groups

**Figure 8:**
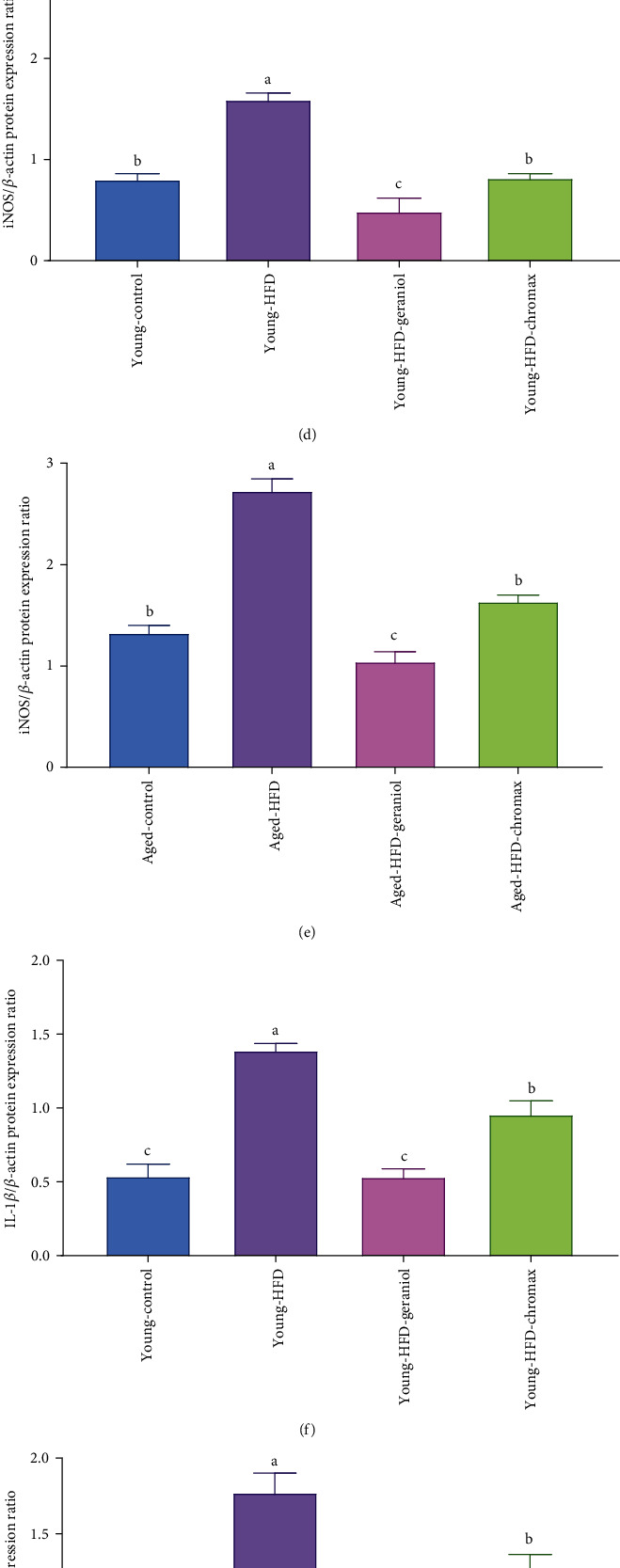
Neuroinflammatory protein expression profile: (a) iNOS/*β*-actin, COX-2/*β*-actin, IL-1*β*/*β*-actin, and p65NF-*κ*B/*β*-actin immunoblot; (b) quantitative analysis of COX-2/*β*-actin in the brain of young rats; (c) COX-2/*β*-actin in the brain of aged rats; (d) iNOS/*β*-actin in the brain of young rats; (e) iNOS/*β*-actin in the brain of aged rats, (f) IL-1*β*/*β*-actin in the brain of young rats, (g) IL-1*β*/*β*-actin in the brain of aged rats, (h) p65NF-*κ*B/*β*-actin in the brain of young rats, and (i) p65NF-*κ*B/*β*-actin in the brain of aged rats. Data are expressed as the mean ± SEM (*n* = 3). Means of the same measured parameter with different letters in each bar (A–D) are significantly different (*p* < 0.01); the largest data value takes the letter (A), and the smallest data value takes the letter (D).

The protective effect of geraniol on the alterations in the protein expression of neuroinflammatory markers COX-2, iNOS, IL-1*β*, and NF-*κ*Bp65 was examined in young and aged experimental rats by western blot analysis, Figure 8(a). The protein expression of iNOS, COX-2, IL-1*β*, and NF-*κ*Bp65 in the brain tissue displayed a significant effect on diet and age. Protein expression of COX-2, iNOS, IL-1*β*, and NF-*κ*Bp65 was changed significantly with age in the control aged NPD-fed rats compared to young NPD-fed rats. Current results revealed that feeding HFD for a long time significantly increased COX-2, iNOS, IL-1*β*, and NF-*κ*Bp65 protein expressions in both aged and young rats compared with those in the control NPD-fed groups. Nevertheless, the protein expression of COX-2 (Figures 8(b) and 8(c)), iNOS (Figures 8(d) and 8(e)), IL-1*β* (Figures 8(f) and 8(g)), and NF-*κ*Bp65 (Figures 8(h) and 8(i)) was considerably suppressed by the geraniol treatment to HFD-fed rats, in particular, young HFD-fed rats more than aged HFD-fed rats when compared with untreated rats, with the greater effect to geraniol-treated groups than chromax.

### 3.13. Histopathological Study of the Brain

**Figure 9:**
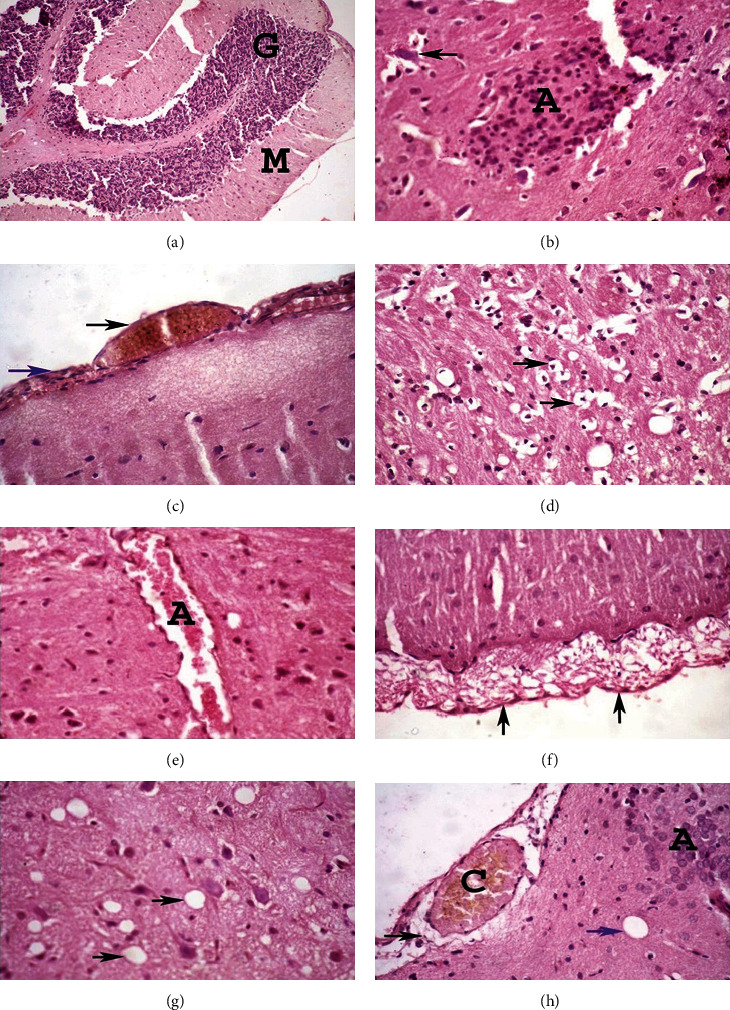
The effects of geraniol on histopathology of the brain in the studied groups. The brain of rats was assessed via hematoxylin-eosin (H&E) staining at 400x magnification: (a) young control group; (b) aged control group; (c) young HFD-fed rats; (d) aged HFD-fed rats; (e) young HFD-fed rats treated with geraniol; (f) aged HFD-fed rats treated with geraniol; (g) young HFD-fed rats treated with chromax; (h) aged HFD-fed rats treated with chromax.

To examine the effect of geraniol and chromax administration on histopathological alterations in the brain of young and aged HFD-fed rats, paraffin sections of studied groups were stained with hematoxylin and eosin (H&E). Photomicrograph of the brain cortex of the young control group (Figure 9(a)) showed normal histoarchitecture, granular layer (G), and molecular layer (M). The photomicrograph of the brain cortex of the aged control group (Figure 9(b)) showed shrunken neurons with satellitosis (arrow) and focal gliosis. Conversely, the photomicrograph of the brain cortex of young HFD-fed rats (Figure 9(c)) showed meningeal hemorrhage (blue arrow) and congestion (black arrow). The photomicrograph of the brain cortex of aged HFD-fed rats (Figure 9(d)) showed dead neurons and perineuronal vacuolation. Moreover, the photomicrograph of the brain cortex of young HFD-fed rats treated with geraniol (Figure 9(e)) showed the congestion of blood vessels (A). The photomicrograph of the brain cortex of aged HFD-fed rats after the administration of geraniol (Figure 9(f)) showed meningeal edema (arrows). Additionally, the photomicrograph of the brain cortex of young HFD-fed rats after treatment with chromax (Figure 9(g)) showed vacuolar degeneration (arrows). In addition, the photomicrograph of the brain cortex of aged HFD-fed rats treated with chromax (Figure 9(h)) showed the congestion of meningeal blood vessels (C), perivascular edema (black arrow), focal gliosis (A), and vacuolation (blue arrow).  Table 5 illustrates the histopathological evaluation grades of different groups' brain lesions using a simple semiquantitative scoring system.

## 4. Discussion

The development and progression of high-mortality diseases are related to lipid metabolic abnormalities. As a consequence of the metabolic alterations associated with a high-fat diet (HFD), neuroinflammation (NI) has been identified to alter brain function by creating varying degrees of cerebral degeneration, culminating in neurodegenerative diseases [42, 43]. Geraniol's antidiabetic, anti-inflammatory, antioxidative, and neuroprotective characteristics [24, 25] prompted us to investigate its impact on NI instigated by HFD. In this study, we noticed that the administration of geraniol could ameliorate HFD-induced weight gain, systemic inflammation, and resistance to insulin.

HFD increased body weight-induced hyperglycemia and dyslipidemia and caused resistance to insulin while reducing the level of insulin-degrading enzyme (IDE). The present research highlighted that the deletion of the liver's IDE worsened the resistance of insulin and glucose intolerance concerning HFD-induced obesity, accompanied by an expansion in plasma insulin levels. Chronic hyperinsulinemia is produced by liver-specific deletion of IDE in the existence of HFD-induced obesity, which causes a decrease in insulin clearance [19]. The study findings are in line with a recent study done by Merino et al. [44]. HFD-induced fatness in mice, according to Merino et al. [44], can be taken as a compensatory mechanism to counter resistance of insulin due to diminished insulin clearance, as predicted from a protease postulated to display the main role in hepatic insulin degradation for decades. Moreover, researchers proved the link concerning low clearance insulin levels and low hepatic IDE activity in people with obesity or type 2 diabetes. This link is critical because IDE destruction can cause increased protein aggregation; this can be a crucial feature in beta cells' pathophysiology of type 2 diabetes and neurons in neurodegenerative illnesses like Alzheimer's and Parkinson's disease [45].

In the current study, the rats showed an upsurge in IDE activity after treatment with geraniol, along with a reduction in glucose and levels of insulin and a reduction in fat accumulation, demonstrating geraniol's antidiabetic efficacy in young and old rats.

In addition, HFD instigated an upsurge in serum C-peptide levels [43]. C-peptide measurements are more trustworthy than blood insulin readings since both are produced from the pancreas at an identical time in equimolar quantities, but C-peptide is not altered by exogenous insulin and is not destroyed by the liver, which can be utilized to represent both insulin's endogenous secretion and pancreatic beta-cell activity [42, 43]. Also, the study signposted that the C-peptide level was significantly elevated in the HFD group in comparison with the control group, while geraniol diminished C-peptide levels, alleviating the state of dyslipidemia.

A previous study by El-Said et al. [46] reported that geraniol alleviated oxidative stress, hyperglycemia, and dyslipidemia in diabetes treatment and metabolic syndrome. Also, geraniol has repeatedly been shown to have an antihyperglycemic impact by altering major carbohydrate metabolism enzymes [23]. It was established that geraniol's antihyperglycemic activity against diabetes complications is mediated by decreasing gluconeogenic enzymes and glucose transporter-2 (GLUT2) [47].

HFD and metabolic disorders can produce a noteworthy increase in circulating triglycerides by altering lipid metabolism [48]. Hence, most studies noticed that triglycerides operated as a lipotoxic factor in the body and were linked to inflammation and IR. IR raised blood triglyceride levels, resulting in diabetic dyslipidemia in diabetic individuals [49]. In insulin-resistant persons, excess triglyceride sources could perform as lipid ligands for apoB, resulting in increased VLDL secretion. In addition, numerous experiments on inflammation found the inverse relation between plasma triglycerides and adiponectin levels [48]. Furthermore, Qiao et al. [49] explained the impact of adiponectin on trigging the breakdown of triglyceride-rich lipoprotein through the stimulation of lipoprotein lipase in adiponectin-overexpressing mice.

Lee et al. [48] discovered that triglyceride levels were linked to white blood cell counts. The elevated macrophage activation enhanced glucose uptake, resulting in the generation of free fatty acids and sterol from glucose-derived carbon and free fatty acid (FFA) uptake. The activation of macrophages also inhibited glucose and free fatty acid oxidation, along with triglyceride lipolysis [48, 50].

According to some studies on the effect of HFD on cognition, stress and obesity are linked to memory and learning problems [51–53]. Similarly, the current data demonstrated a remarkable diet and age effect on the distance swum and escape latency in the Morris water maze training, revealing interrupted behavioral flexibility in the HFD-fed animals. Moreover, an obesogenic diet could enhance the free radical formation and oxidative stress in rodent brains [54]. It has been extensively recognized that one of the primary causes of cognitive impairment is severe oxidative stress. Numerous studies found a direct correlation between high cholesterol levels and the occurrence of Alzheimer's disease [55]. According to clinical investigations, there is a connection between the brain and peripheral cholesterol, and the serum cholesterol level regulates the amount of cholesterol in the brain [56]. Rats with hypercholesterolemia impair cholinergic function and have poor memory [57]. Notably, the ameliorative effects of geraniol on cognitive performance are probably caused by modifications in lipid profiles. In addition, geraniol has been recently recommended as a viable therapeutic agent for enhancing cognitive function and neurotoxicity produced in rats [58, 59].

Increased inflammatory mediators and reduced adiponectin also showed low-grade systemic inflammation (LGSI) in HFD-induced obese rats. The adipose tissue, which is subjected to cellular composition changes and macrophage infiltration, causing the excretion of a multiplicity of adipokines and cytokines, is one of the major tissues implicated in LGSI during obesity. These derangements are linked to the initiation and progression of obesity-related metabolic complications [42, 49]. Obesity-induced inflammation is triggered by the secretion of proinflammatory adipokines by adipose tissue, particularly visceral adipose tissue, including leptin, along with a diminution in the synthesis of anti-inflammatory adiponectin. Correspondingly, some studies signposted that the classes of adipokines, specifically FFA, adiponectin, and leptin, are significant biomarkers and have a vital role in the pathology of obesity [49]. The study demonstrated that HFD-induced obesity in rats had a substantial increase in serum leptin and declined adiponectin level, whereas the animal group treated with geraniol significantly improved the adiponectin level and lessened the serum leptin level.

Chronic hyperglycemia, peripheral IR, dyslipidemia, and/or prolonged whole-body inflammation are major contributors to neuronal redox imbalance and oxidative damage [42], and there is a bidirectional interaction between peripheral and central inflammation [60, 61]. Reactive oxygen species (ROS) propagation was noticed to be high in brain tissue endothelial cells, pericytes, and astrocytes [62, 63], presumably due to excess glucose and fatty acids. In addition, long-term HFD was known to trigger oxidative stress and neuroinflammation in the brain, as evidenced by glial activation and cytokine production [2]. Hyperglycemia also causes the upsurge of inflammatory cytokines by trigging ROS generation via glucose autoxidation, the polyol pathway, and nonenzymatic glycation, blocking glycolytic enzymes and oxidizing cell components [64].

Previous studies proved that triggered oxidative stress and altered apoptosis contribute to the pathogenesis of neurodegenerative diseases [42]. Free radicals are concerned with the progression and development of cognitive deficits by interrupting synaptic transmission, mitochondrial function, neuroinflammation, and axonal transport. These factors are related to neuronal loss in Alzheimer's and other dementia diseases [65]. Furthermore, mitochondria are part of the pathophysiology of a wide range of neurodegenerative disorders. Mitochondria are the major organelles in cells that produce ATP through oxidative phosphorylation, respond to oxidative stress, and create other important chemicals. They produce redox enzymes, which are essential to transfer electrons from one substrate to another. Deficiencies in this process can lead to the generation of ROS. Due to its enormous energy demands, the central nervous system relies heavily on mitochondrial activity [66]. Neurodegenerative disease and brain dysfunction can be brought out by the mutations in mitochondrial DNA and the development of ROS. Similarly, mitochondrial dysfunction is linked to a diversity of neurodegenerative disorders [67].

Our study indicated that the oral geraniol treatment not only reduced the weight of the body and improved lipid serum levels in HFD rats but also dramatically reduced brain oxidative stress by downregulating MDA while upregulating superoxide dismutase (SOD), catalase (CAT), glutathione-S-transferase (GST), and glutathione (GSH) levels.

The antioxidant features of geraniol have been well established, including free radical scavenging and suppression of lipid peroxidation (LPO), besides enhancing antioxidant capacity and neuroprotection [24]. Elguindy et al. [24] reported that geraniol restores the redox imbalance caused by DENA-induced oxidative stress in the brain. Noticeably, rats given geraniol had lower LPO levels in their kidneys and brains, but rats given HFD had higher LPO levels. This finding highlighted geraniol's high antioxidant effect, which was inconsistent with the previous works of Hosseini et al. [68].

Moreover, Farokhcheh et al. [58] discovered that geraniol could boost glutathione peroxidase activity (GPX) and SOD, besides functioning as a free radical scavenger, causing a decrease in ROS, all of which would lower MDA, the major result of lipid peroxidation.

Geraniol has been proven to lower the intracellular concentration of oxidants after treatment [68, 69]. Furthermore, geraniol enhances the activity of several antioxidant enzymes to regulate oxidative stress damage by stimulating the nuclear factor erythroid 2-related factor 2 (Nrf2) [69]. Previous research demonstrated that geraniol reduces iNOS, COX-2, and NF-*κ*B, simultaneously improving CAT, GPX, GSH reductase, and SOD levels [42, 58].

Indoleamine 2,3-dioxygenase (IDO1) is primarily located in microglia, which suggests that it can have a role in a range of neurodegenerative diseases, e.g., Alzheimer's and Parkinson's, plus mental disorders, including depression and schizophrenia [70]. This finding could be due to its expression in CNS cells, such as glial cells, dendritic cells (DCs), macrophages, antigen-presenting cells (APCs), endothelial cells, and vascular smooth muscle cells [71]. When the CNS is under inflammation, IDO1 directs the kynurenine pathway (KDP) degradation to trigger the transformation of Trp into neurotoxic compounds, leading to neurotoxicity by producing adequate quantities of ROS, persuading neuronal apoptosis [70]. These neurotoxins can correspondingly prompt lipid peroxidation, upregulate iNOS expression, and reduce SOD activity, causing mitochondria dysfunction and activating a positive feedback loop expression of IDO1 [72, 73].

Furthermore, cytokines' release, such as tumor necrosis factor (TNF) and interleukin-6 (IL-6), is elevated, which is produced from the activated macrophages of the adipose tissue during obesity [74]. TNF-*α* is related to the development of obesity-related insulin resistance, whereas IL-6 promotes lipolysis [75]. Wang et al. [2] indicated that HFD-induced obese mice displayed dramatically reinforced levels of various inflammatory transcript factors, such as cyclooxygenase-2 (COX-2), inducible nitric oxide synthase (iNOS), and nuclear factor kappa B (NF-*κ*B) in the brain. Peripheral inflammation was detected following 3 months of HFD. Also, peripheral cytokines can affect the brain, causing local cytokine production [76]. In the current study, we discovered that a long-term HFD increased TNF-*α*, IL-6, and IL-1 levels in the brain, which were suppressed by long-term geraniol therapy, designating that geraniol has an anti-inflammatory impact.

Increased FFA and hyperglycemia-induced oxidative stress were related to changes in AChE activity [42]. Acetylcholine functions on the “cholinergic anti-inflammatory pathway,” inhibiting the creation of inflammatory cytokines, like IL-6 and TNF; it, hence, increases brain acetylcholinesterase activity and aggravates local and systemic inflammatory processes [77]. In pathological situations, AChE activity can be employed as a measure of central cholinergic status, resulting in increased AChE activity [78].

In this study, we discovered that HFD caused oxidative stress, gliosis, necrosis, and memory loss. However, due to its antioxidant and anti-inflammatory qualities, geraniol therapy increased learning and memory performance. Herbal therapy has recently emerged as a viable treatment option for a multiplicity of neurological illnesses. Geraniol was informed to have neuroprotective properties in an animal model with Parkinson's disease [58]. Geraniol administration also suppresses AChE activity in diabetic rats, contributing to increased cholinergic neurotransmission and improved cognitive abilities [79]. In other research, geraniol therapy reduced the neurotoxicity and mental deficits instigated by an acrylamide model in Drosophila melanogaster [80]. With the reduction in brain AChE activity of diethylnitrosamine-induced neuroinflammation, geraniol treatment has already been described [24], which supports our observations.

The exitance of microgliosis and astrogliosis and an aberrant rise of the astrocytes in the HFD rat's hippocampus are corroborated by our light microscopy studies. Elevated iNOS expression [42] occurs in obesity-induced hippocampal neuroinflammation, resulting in an abundant source of NO, which is linked to nitrosative stress, a potent obstructer of the insulin-signaling pathway in obesity [81].

An ascent in the hippocampal iNOS expression of young and aged HFD rats was achieved in our study, which was harmonized in which an HFD was able to trigger an upsurge in hippocampal iNOS in C57BL/6J male mice [82]. Furthermore, a link between the inflammatory cytokines and a deteriorated hippocampal acetylcholine occurred, in which inflammatory cytokines could elevate hippocampal neuronal AChE expression [42]. In the study, an increased hippocampal AChE expression was spotted in obese rats linked with hippocampal inflammation instigated by hyperglycemia and oxidative stress. We found that geraniol treatment reduced hippocampal AChE expression in both young and old HFD rats, confirming its power to decrease oxidative stress and neuroinflammation. We also discovered that geraniol had wide anti-inflammation, significantly reducing iNOS expression in HFD-geraniol rat hippocampi.

We demonstrated that age and HFD promoted neurotoxicity in the cortical and hippocampus areas, which is consistent with prior reports. According to the histological investigation of the brain, geraniol therapy dramatically restored neuronal morphology and survival in the brains of treated rats. Previously, geraniol was shown to have neuroprotective properties in an animal model of Parkinson's disease [58]. Recent studies demonstrated that geraniol treatment markedly boosted tyrosine hydroxylase, brain-derived neurotrophic factor (BDNF), and glial-derived neurotrophic factor (GDNF) and decreased apoptosis [26, 58, 59].

## 5. Conclusions

We conclude that geraniol can prevent the development of neuroinflammation and toxicity in young and old rats due to metabolic changes caused by HFD by exerting its hypoglycemic effect and restoring pancreatic function, besides preventing IR. The administration of geraniol upgraded the level of IDE, ameliorated the adipokine levels, and alleviated dyslipidemia. Furthermore, geraniol's anti-inflammatory and antioxidant properties were key contributors to combating general oxidative stress and low-grade systemic inflammation primarily concerned with HFD. Geraniol administration with a dose of 200 mg/kg was associated with an outstanding decline in gene expressions and protein levels of key contributors to neuroinflammation, as well as a reduction in the structural damage to the brain caused by HFD. Therefore, geraniol supplementation in the elderly may be a noninvasive approach to reducing chronic neuroinflammation and neurotoxicity-related age and diet.

## Figures and Tables

**Table:** (a) Effect of geraniol administration on blood parameters of aged and young HFD-fed rats

Groups	ALT (U/l)	AST (U/l)	ALP (U/l)	ALB (g/dl)	T. protein (g/dl)	T. bilirubin (*μ*mol/l)	Creatinine (mg/dl)	Urea (mg/dl)
Young control	37.64 ± 1.17^c^	99.64 ± 1.01^c,d^	91.57 ± 0.71^b^	3.52 ± 0.12^a,b^	7.12 ± 0.17^a,b^	0.41 ± 0.01^b^	0.43 ± 0.02^f^	10.40 ± 0.51^c^
Aged control	30.46 ± 1.02^c^	100.22 ± 1.66^c,d^	93.66 ± 0.83^b^	3.62 ± 0.30^a,b^	6.56 ± 0.08^a,b,c^	0.36 ± 0.01^b^	0.52 ± 0.02^e,f^	18.80 ± 1.59^b,c^
Young HFD	141.26 ± 0.41^b^	128.02 ± 2.18^b^	85.76 ± 1.65^b^	3.80 ± 0.14^a,b^	6.12 ± 0.05^c^	0.42 ± 0.02^b^	1.55 ± 0.06^b^	22.40 ± 1.29^b^
Aged HFD	184.28 ± 2.25^a^	239.88 ± 3.11^a^	190.92 ± 2.96^a^	2.46 ± 0.29^b^	4.36 ± 0.08^d^	1.82 ± 0.07^a^	1.87 ± 0.04^a^	47.80 ± 1.96^a^
Young HFD-geraniol	33.74 ± 0.99^c^	103.26 ± 2.33^c^	92.16 ± 0.87^b^	3.06 ± 0.25^a,b^	7.12 ± 0.27^a,b^	0.43 ± 0.01^b^	0.67 ± 0.02^d,e^	15.40 ± 2.01^b,c^
Aged HFD-geraniol	35.08 ± 1.17^c^	94.28 ± 1.18^c,d^	73.50 ± 1.17^c^	4.00 ± 0.17^a^	7.22 ± 0.06^a^	0.43 ± 0.01^b^	0.80 ± 0.04^c,d^	18.20 ± 0.37^b,c^
Young HFD-chromax	32.62 ± 1.20^c^	87.28 ± 0.88^d^	71.08 ± 1.66^c^	4.40 ± 0.13^a^	6.32 ± 0.10^b,c^	0.43 ± 0.01^b^	0.68 ± 0.02^c,d,e^	16.80 ± 0.37^b,c^
Aged HFD-chromax	36.88 ± 1.38^c^	93.18 ± 2.19^c,d^	72.88 ± 1.29^c^	3.90 ± 0.16^a^	6.44 ± 0.05^a,b,c^	0.39 ± 0.01^b^	0.91 ± 0.03^c^	11.80 ± 0.37^c^

Values are tabulated as the mean ± SEM (*n* = 8). Means of the same parameter with different letters (a–e) in each column are significantly different (*p* < 0.01); the largest values take the letter (a), and the smallest values take the letter (e).

**Table:** (b) Effect of geraniol administration on lipid profile parameters of aged and young HFD-fed rats

Groups	Cholesterol (mg/dl)	TG (mg/dl)	LDL-c (mg/dl)	HDL-c (mg/dl)
Young control	98.20 ± 0.66^d^	70.60 ± 2.50^e^	55.00 ± 1.52^f^	74.60 ± 3.54^a^
Aged control	105.20 ± 1.88^d^	84.60 ± 1.81^e^	62.40 ± 1.03^e,f^	60.20 ± 1.91^b^
Young HFD	229.60 ± 7.95^b^	191.80 ± 3.40^b^	107.60 ± 1.54^b^	37.80 ± 1.88^d,e^
Aged HFD	390.00 ± 10.95^a^	368.80 ± 11.26^a^	138.20 ± 1.50^a^	24.80 ± 0.66^e^
Young HFD-geraniol	107.60 ± 2.38^d^	126.20 ± 2.08^d^	64.80 ± 1.24^e,f^	59.20 ± 1.93^b^
Aged HFD-geraniol	171.60 ± 6.14^c^	145.20 ± 1.46^c,d^	81.40 ± 1.33^c,d^	51.60 ± 0.81^b,c^
Young HFD-chromax	130.60 ± 2.75^d^	146.00 ± 2.74^c,d^	71.80 ± 1.24^d,e^	57.20 ± 2.62^b^
Aged HFD-chromax	214.20 ± 3.25^b^	157.20 ± 1.74^c^	89.80 ± 3.77^c^	41.20 ± 0.80^c,d^

Values are tabulated as the mean ± SEM (*n* = 8). Means of the same parameter with different letters (a–e) in each column are significantly different (*p* < 0.01); the largest values take the letter (a), and the smallest values take the letter (e).

**Table 2:** Effect of geraniol administration on blood parameters of aged and young HFD-fed rats.

Groups	FBG (mg/dl)	Insulin (*μ*IU/ml)	HOMA-IR	C-peptide (pg/ml)	IDE (ng/mg protein)
Young control	79.60 ± 3.41^e^	4.64 ± 0.08^c^	0.90 ± 0.03^c^	309.17 ± 8.50^e^	36.10 ± 0.15^a^
Aged control	100.00 ± 3.27^d,e^	4.98 ± 0.06^c^	1.20 ± 0.08^c^	572.50 ± 22.11^e^	27.12 ± 0.21^b^
Young HFD	156.80 ± 4.61^b^	16.37 ± 0.52^b^	6.30 ± 0.04^b^	2259.17 ± 45.52^b^	12.00 ± 0.31^d^
Aged HFD	223.20 ± 3.61^a^	22.99 ± 1.14^a^	12.67 ± 0.23^a^	2779.17 ± 113.82^a^	9.24 ± 0.22^d^
Young HFD-geraniol	100.20 ± 1.16^d^	4.58 ± 0.02^c^	1.13 ± 0.07^c^	1042.50 ± 33.58^d^	29.16 ± 0.18^b^
Aged HFD-geraniol	125.00 ± 2.21^c^	4.92 ± 0.06^c^	1.52 ± 0.09^c^	1442.50 ± 13.54^c^	17.09 ± 0.30^c^
Young HFD-chromax	115.80 ± 1.62^c,d^	5.19 ± 0.04^c^	1.53 ± 0.10^c^	1532.50 ± 11.79^c^	27.17 ± 0.39^b^
Aged HFD-chromax	132.40 ± 2.84^c^	5.89 ± 0.13^c^	1.93 ± 0.13^c^	1745.83 ± 25.50^c^	19.08 ± 0.44^c^

Values are tabulated as the mean ± SEM (*n* = 8). Means of the same parameter with different letters (a–e) in each column are significantly different (*p* < 0.01); the largest values take the letter (a), and the smallest values take the letter (e).

**Table 3:** Effect of geraniol administration on brain oxidative stress markers and AChE activity of aged and young HFD-fed rats.

Groups	TBARS (*μ*mol/mg protein)	XO (*μ*mol/h/mg protein)	NO (*μ*mol/mg protein)	AChE (mmol/min/mg protein)
Young control	16.86 ± 1.49^c^	5.70 ± 0.38^c^	20.08 ± 0.93^c^	1.57 ± 0.07^c^
Aged control	43.98 ± 1.14^c^	11.00 ± 2.92^c^	45.94 ± 4.53^b,c^	3.13 ± 0.19^c^
Young HFD	91.76 ± 3.82^b^	64.60 ± 5.02^b^	112.45 ± 7.86^b^	15.52 ± 0.80^b^
Aged HFD	173.47 ± 11.18^a^	87.00 ± 4.01^a^	273.60 ± 31.65^a^	23.48 ± 2.09^a^
Young HFD-geraniol	20.13 ± 1.46^c^	1.28 ± 0.18^c^	10.55 ± 1.12^c^	1.24 ± 0.06^c^
Aged HFD-geraniol	29.78 ± 1.18^c^	8.00 ± 1.79^c^	19.25 ± 2.25^c^	2.37 ± 0.08^c^
Young HFD-chromax	28.12 ± 1.21^c^	9.00 ± 2.26^c^	31.30 ± 3.52^c^	2.88 ± 0.18^c^
Aged HFD-chromax	34.98 ± 0.62^c^	11.40 ± 2.73^c^	43.65 ± 4.09^b,c^	3.50 ± 0.07^c^

Values are tabulated as the mean ± SEM (*n* = 8). Means of the same parameter with different letters (a–c) in each column are significantly different (*p* < 0.01); the largest values take the letter (a), and the smallest values take the letter (c).

**Table 4:** Effect of geraniol administration on brain antioxidant status of aged and young HFD-fed rats.

Groups	GST (*μ*mol/min/mg protein)	SOD (*μ*mol/min/mg protein)	CAT (U/mg protein)	GSH (*μ*mol/mg protein)
Young control	7.34 ± 0.27^c,d^	8.90 ± 1.21^d^	1.72 ± 0.07^c,d,e^	10.92 ± 0.50^c^
Aged control	8.23 ± 0.41^c^	20.25 ± 5.33^b,c^	2.55 ± 0.14^c^	14.72 ± 0.82^c^
Young HFD	3.14 ± 0.24^e^	2.46 ± 0.65^d^	1.25 ± 0.07^d,e^	2.21 ± 0.32^d^
Aged HFD	3.88 ± 0.50^d,e^	2.17 ± 1.10^d^	1.06 ± 0.32^e^	0.62 ± 0.11^d^
Young HFD-geraniol	29.85 ± 0.68^a^	90.21 ± 3.92^a^	4.74 ± 0.19^a^	32.03 ± 2.10^a^
Aged HFD-geraniol	14.16 ± 0.85^b^	40.52 ± 2.59^b^	2.87 ± 0.11^b,c^	23.46 ± 1.22^a,b^
Young HFD-chromax	21.48 ± 0.82^a^	36.32 ± 2.40^b,c^	3.97 ± 0.29^a,b^	31.04 ± 2.14^a^
Aged HFD-chromax	14.40 ± 0.59^b^	17.01 ± 4.56^c,d^	2.39 ± 0.07^c,d^	17.98 ± 1.23^b,c^

Values are tabulated as the mean ± SEM (*n* = 8). Means of the same parameter with different letters (a–c) in each column are significantly different (*p* < 0.01); the largest values take the letter (a), and the smallest values take the letter (c).

**Table 5:** The semiquantitative grading system of brain histopathological alterations.

Scored brain lesions	Incidence^1^ and severity^2^ of histopathological lesions																											
	Aged control				Young HFD				Aged HFD				Young HFD-geraniol				Aged HFD-geraniol				Young HFD-chromax				Aged HFD-chromax			
	(-)	(+)	(++)	(+++)	(-)	(+)	(++)	(+++)	(-)	(+)	(++)	(+++)	(-)	(+)	(++)	(+++)	(-)	(+)	(++)	(+++)	(-)	(+)	(++)	(+++)	(-)	(+)	(++)	(+++)
(1) Congestion of blood vessels (meningeal and cortical)	2	2	3	1	1	1	3	3	1	1	3	3	4	3	1	0	4	1	3	0	3	3	2	0	1	1	4	2
(2) Perivascular cuffing	1	1	2	3	0	1	2	4	1	1	1	5	3	2	2	1	3	0	3	2	3	1	1	3	0	1	3	4
(3) Vacuolar degeneration	2	1	4	1	1	1	3	3	0	2	2	4	5	1	2	0	4	2	1	1	3	1	3	1	1	1	3	3
(4) Meningeal edema	2	1	3	2	1	1	2	2	1	1	4	2	6	1	1	0	5	1	1	1	3	3	2	0	1	1	2	4
(5) Neuronal shrinkage and satellitosis	3	3	1	1	1	2	2	3	2	0	3	3	4	2	0	2	3	3	1	1	4	1	3	0	1	2	2	3
(6) Focal gliosis	2	1	3	2	1	1	2	3	1	1	2	4	5	3	0	0	3	3	0	2	3	2	2	2	1	1	3	3
(7) Meningeal hemorrhage	4	1	1	2	1	1	4	2	1	0	5	2	7	1	0	0	6	2	0	0	5	0	2	1	1	1	2	4

## Data Availability

Data produced from this work and presented in this manuscript is available upon request from the corresponding author.

## Abbreviations

ACh: Acetylcholine
AChE: Acetylcholinesterase
apoB: Apolipoprotein B
CAT: Catalase
CNS: Central nervous system
COX-2: Cyclooxygenase-2
DENA: Diethylnitrosamine
DTNB: 5,5-Dithiobis(2-nitrobenzoic acid)
GLUT2: Glucose transporter-2
GPX: Glutathione peroxidase
GSH: Reduced glutathione
GST: Glutathione-S-transferase
HFD: High-fat diet
HOMA-IR: Homeostatic model assessment index
IDO1: Indoleamine 2,3-dioxygenase 1
IL: Interleukin
iNOS: Induced nitric oxide synthase
IDE: Insulin-degrading enzyme
LGSI: Low-grade systemic inflammation
LPO: Lipid peroxidation
MDA: Malondialdehyde
NF-*κ*B: Nuclear factor kappa B
NO: Nitric oxide
NPD: Normal pellet diet
Nrf2: Nuclear factor erythroid 2-related factor 2
ROS: Reactive oxygen species
SGLT-1: Sodium glucose cotransporter
SOD: Superoxide dismutase
TBARS: Thiobarbituric acid reaction
TNF-*α*: Tumor necrosis factor-alpha
XO: Xanthine oxidase.
